# The Uptake and Factors Associated with Mastectomy among Chinese Women with Breast Cancer: A Retrospective Observational Study

**DOI:** 10.31557/APJCP.2021.22.5.1599

**Published:** 2021-05

**Authors:** Jing Liu, Dongmei Guo, Sharyn Hunter, Regina Lai Tong Lee, Jiemin Zhu, Sally Wai-Chi Chan

**Affiliations:** 1 *School of Nursing and Midwifery, University of Newcastle, New South Wales, Australia. *; 2 *Department of Breast Surgery, Zhongshan Hospital Xiamen University, Xiamen, China. *; 3 *Department of Nursing, School of Medicine, Xiamen University, Xiamen, China. *; 4 *President’s Office, Tung Wah College, Hong Kong, China. *

**Keywords:** Breast neoplasms, mastectomy, data analysis, early detection of cancer

## Abstract

**Objective:**

There are limited data concerning the use of mastectomy and associated factors in China in recent years. This study aimed to investigate the uptake of mastectomy and determine the associations between patients’ characteristics and mastectomy among Chinese women with breast cancer.

**Methods:**

A retrospective analysis of female breast cancer cases from January 2015 to December 2019 from a tertiary hospital was conducted. Socio-demographic data, clinical data, and surgery types were collected by reviewing the medical record system. Chi-square test, Fisher’s exact test and multivariate logistic regression analysis were used to determine correlations of patients’ characteristics with mastectomy.

**Results:**

A total of 1,171 women with breast cancer were identified, and 76.60% of them underwent a mastectomy. The mastectomy rates showed an increase from 70.62% in 2015 to 86.87% in 2017 and then dropped to 71.91% in 2019. Women undergoing mastectomy were older and were more likely to be married and have at least one child. They had an advanced cancer stage, larger tumour size, and more positive lymph nodes and were HER-2-overexpressing. Older age, larger tumour size (2-5 cm), higher cancer stages (stage 2- stage 3) and being positive for HER-2 were the four independent variables that significantly predicted the uptake of mastectomy.

**Conclusions:**

Our results showed a wide application of mastectomy in China and uncovered the factors associated with mastectomy uptake from a single-centre experience. Findings suggested the potential overuse of mastectomy among women with early-stage breast cancer, and highlighted the significance of promoting cancer screening in China. Findings could be also used to develop relevant provisions and interventions to facilitate breast cancer treatment decision-making and screening planning.

## Introduction

Mastectomy is the total removal of a cancer-affected breast. It is an appropriate treatment for most types of breast cancer. With the progress of treatment procedures, mastectomy has been used less in many Western countries, such as the United States (US) (Habermann et al., 2010) and European countries (Garcia-Etienne et al., 2012). However, mastectomy rates are stably high in Asian regions, such as Malaysia (Wong et al., 2019), Singapore (Chan et al., 2015), Saudi Arabia (Al-Gaithy et al., 2019) and China (Huang et al., 2016), with more than half of breast cancer survivors that have had a mastectomy in these Asian countries. 

As the most commonly diagnosed cancer among Chinese women, the incidence of breast cancer in China has substantially increased in recent years (Chen et al., 2016). It is expected that approximately 2.5 million Chinese women aged 55-69 years will be diagnosed with breast cancer by 2021 (Linos et al., 2008). In the existing literature, there is a lack of national estimates about how many mastectomy surgeries are conducted per year. The most recent study specific to the uptake of mastectomy in China was published in 2016, reporting that more than 80% of 18,502 patients with breast cancer received a mastectomy during 1999 and 2013 at a breast cancer centre in Shanghai. The findings from this study suggested significant associations between older age and advanced cancer stage with increasing uptake of mastectomy (Huang et al., 2016). Another multi-centre survey among 4,211 patients with breast cancer was conducted in 2015, reporting high mastectomy rates ranging from 88.12% to 98.47% during 1999 and 2008 (Zhang et al., 2015). They found married women with older age, lower education level, and an advanced cancer stage preferred mastectomy instead of breast-conserving surgery (BCS; lumpectomy with radiotherapy). Although there were regional variations in the mastectomy rates, the proportion of patients treated with a mastectomy in China was remarkably higher than the global average (generally lower than 50%). However, these studies concerning the prevalence of mastectomy in China were all conducted at least three years ago.

Cancer screening helps to detect breast cancers early, so it can avoid mastectomies from being necessary in many cases (Harding et al., 2019). In recent years, there are great advances in cancer screening and surgical procedures for breast cancer treatment in China, particularly after a national consensus on the education for breast cancer prevention was reached in 2017. However, there lacked recent data on changes in mastectomy use and associated factors with mastectomy. This study aimed to determine the trend in mastectomy rates (from January 2015 to December 2019), as well as to investigate any associated factors of mastectomy in China. 

## Materials and Methods

*Study Design*

This study employed a retrospective cross-sectional design analyzing breast cancer cases from a single centre in China from January 2015 to December 2019. The data were sourced from the electronic medical record system (EMRS) in Zhongshan Hospital Xiamen University (ZSXM), a tertiary general hospital in eastern China. The data contained in the EMRS was entered by surgeons and nurses who had training in data entry for the breast cancer registry based on the hospital’s guidelines. The records are evaluated annually for completeness and accuracy to ensure data quality.

Ethical approval for this study was obtained from the Human Research Ethics Committee of the University of Newcastle (No. H-2020-0004). Permission for using these secondary data was obtained from the hospital. 

*Data*

Female patients who were diagnosed with breast cancer and received treatments in this hospital from 1^st ^January 2015 to 31^st^ December 2019 were included. All female breast cancer cases during the audit period were retrospectively analyzed to avoid selection bias. No identifiable personal data were collected. Three types of patient information were retrospectively collected from the EMRS, including: 

1) Socio-demographic characteristics (age, ethnicity, marital status, fertility history, employment status, healthcare costs, living area);

2) Clinical characteristics (cancer stage, tumour size, the presence of lymph node invasion, histology, the presence of hormone receptors, biological subtypes, a family history of breast cancer);

3) Types of surgical procedures.

In this study, we considered mastectomy to include simple mastectomy, modified radical mastectomy, total mastectomy or skin/areola/nipple-sparing mastectomy, not otherwise specified (Al-Gaithy et al., 2019). The rate of mastectomy was defined as the percentage of mastectomy among all breast cancer cases. The tumour size was defined as the maximum pathological tumour diameter. Lymph node invasion was assessed using the number of positive lymph nodes. Breast cancers were categorized into four biological subtypes based on the presence of the estragon receptor (ER), progesterone receptor (PR), and human epidermal growth factor receptor 2 (HER-2) status. Two researchers independently collected the data from the EMRS. Data quality was ensured by a full discussion among the researchers. 

*Data analysis*

The data were analyzed using SPSS version 26 software. The data were summarized using descriptive statistics, such as the mean and percentage. The chi-square test was used to compare patient’s characteristics between mastectomy and non-mastectomy groups. Fisher’s exact test was used for categorical variables in which the number of categories was less than five. Statistically significant variables were then inputted into the multivariate logistic regression analysis to determine their predictive effects on the use of mastectomy. Patients with missing data were excluded from the analysis. Two-tailed P values were used. P <0.05 was considered to be statistically significant.

## Results

*Mastectomy Rates*

A total of 1,171 women with breast cancer received treatments in this hospital. Among these, 897 patients (76.60%) underwent a mastectomy, and only 27 patients (2.31%) had immediate reconstruction after mastectomy. There were 166 patients (14.18%) who underwent a BCS. A total of 108 patients (9.22%) did not receive any surgical treatments.

The numbers of mastectomy cases were presented in Table 1. The data showed that the mastectomy rate increased from 70.62% in 2015 to 86.87% in 2017, following a drop to 71.91% in 2019. 

*Patient’s Characteristics*

The patients’ ages ranged from 22 to 90 years old, and their mean age was 50.69 years. Among the total 1,171 women, the majority were of Han ethnicity (n=1,164, 99.40%), which is the major ethnic group in China, married (n=1,129, 96.41%), had at least one child (n=1,111, 94.88%) and being employed (n=740, 63.28%). Most women (n=957, 81.73%) had their health care costs fully or partially covered by health insurance. 

The mean tumour size was 3.08 cm. Among the total 1,171 women, 836 (71.39%) had early-stage breast cancer (ESBC; stage 0-2) and 81.10% of women with ESBC underwent a mastectomy. The most common pathological histology was invasive ductal (n=10,10, 86.25%). Approximately 13.07% (n=153) of all women had a more aggressive triple-negative breast cancer (ER−/PR−/HER-2−). Over half of the patients had no positive lymph nodes (n=656, 56.02%). Few women (n=11, 0.94%) reported a known family history of breast cancer. Socio-demographic characteristics and clinical characteristics of women with mastectomy and non-mastectomy were shown in Table 2.

*Factors Related to the Uptake of Mastectomy*

As Table 2 showed, there were significant differences in age (*χ*^2^=18.700, p =0.000), marital status *(χ*^2^=14.334, p =0.001), fertility history (*χ*^2^=14.021, p =0.000), tumour size (*χ*^2^=27.003, p =0.000), histology (*χ*^2^= 11.652, p =0.016), cancer stage (*χ*^2^=109.386, p =0.000), lymph node invasion (*χ*^2^=12.986, p = 0.001), and expression of HER-2 (*χ*^2^=3.948, p =0.047) between the mastectomy and non-mastectomy groups. Compared with patients without a mastectomy, those undergoing a mastectomy were older, married, had at least one child, a higher cancer stage at diagnosis, a larger tumour, more positive nodes and were positive for HER-2. No significant differences were found for the other patient characteristics. 

Using a multivariate logistic regression model adjusted for these correlated variables (age, marital status, fertility history, cancer stage, tumour size, histology, lymph node invasion, and the expression of HER-2), older age, tumour size of 2-5 cm, stage 2-stage 3 cancers, and positive for HER-2 were significant independent predictors of mastectomy uptake (see Table 3).

**Table 1:** The Number of Mastectomy Cases and Mastectomy Rates among Women with Breast Cancer in ZSXM, China, from 2015 to 2019 (Total N=1171)

	Years, mastectomy cases (breast cancer cases)				
	2015	2016	2017	2018	2019
Stage 0	8 (11)	11 (19)	16 (19)	24 (32)	16 (29)
Stage 1	19 (33)	30 (44)	34 (40)	56 (67)	41 (53)
Stage 2	62 (75)	78 (86)	88 (93)	96 (114)	99 (121)
Stage 3	29 (34)	34 (41)	30 (34)	44 (49)	52 (65)
Stage 4	6 (13)	4 (14)	1 (7)	5 (11)	4 (16)
Unknown	1 (11)	1 (7)	3 (5)	2 (13)	3 (15)
Mastectomy cases, n	125	158	172	227	215
Breast cancer cases, n	177	211	198	286	299
Mastectomy rates^a^, %	70.62	74.88	86.87	79.37	71.91

^a^, The number of mastectomy cases divided by the total number of breast cancer cases in each year.

**Table 2:** Socio-demographic and Clinical Characteristics among Women with Breast Cancer in ZSXM, China, from 2015 to 2019 (Total N=1171)

Variables	Total			Mas			Non-Mas ^a^				*P-value*
	N			n	%^b^		n			%^b^	
Age Groups, Years											<0.001*
22-39	184			108	58.70		76			41.30	
40-59	713			553	77.56		160			22.44	
60+	274			209	76.28		65			23.72	
Ethnicity											0.669
Han	1164			892	76.63		272			23.37	
Others (She, Hui, Man, and Tujia)	7			5	71.43		2			28.57	
Marital Status											<0.001*
Married	1129			873	77.33		256			22.67	
Single	32			15	46.88		17			53.13	
Divorced	8			7	87.50		1			12.50	
Widowed	2			2	100.00		0			0	
Employment Status											0.370
Employed	740			559	75.54		181			24.46	
Unemployed	212			170	80.19		42			19.81	
Retired	219			168	76.71		51			23.29	
Fertility History											<0.001*
No Children	60			34	56.67		26			43.33	
One or More Children	1111			863	77.68		248			22.32	
Living Area											0.262
Inner City	714			539	75.49		175			24.51	
Outside the City	457			358	78.34		99			21.66	
Treatment Costs											0.157
Out-of-pocket Money	214			156	72.90		58			27.10	
Fully or Partially Covered by Insurance	957			741	77.43		216			22.57	
Tumour Size (cm)											<0.001*
<2	229			168	73.36		61			26.64	
2–5	736			644	87.50		92			12.50	
>5	92			80	86.96		12			13.04	
Cancer Stage ^c^											<0.001*
Stage 0	110			75	68.18		35			31.82	
Stage 1	237			180	75.95		57			24.05	
Stage 2	489			423	86.50		66			13.50	
Cancer Stage ^c^											<0.001*	
Stage 3		223	189			84.75		34	15.25			
Stage 4		61	20			32.79		41	67.21			
Histology											0.011*	
Non-invasive		110	75			68.18		35	31.82			
Invasive ِDuctal		1010	785			77.72		225	22.28			
Invasive Lobular		19	10			52.63		9	47.37			
Mixed Ductal/Lobular		5	5			100.00		0	0.00			
Others (Papillary, Mucinous, Medullary, Tubular, and Paget Disease)		27	22			81.4		5	18.52			
Biological Subtypes ^c^											0.263	
Luminal A		642	497			77.41		145	22.59			
Luminal B		176	147			83.52		29	16.48			
Triple-negative		153	118			77.12		35	22.88			
HER-2-enriched		151	123			81.46		28	18.54			
Lymph Node Invasion ^c^											0.002*	
0 (Non-invasion)		656	536			81.71		120	18.29			
1-9		316	280			88.61		36	11.39			
10+		76	71			93.42		5	6.58			
ER ^c^											0.949	
Negative		323	255			78.95		68	21.05			
Positive		801	631			78.78		170	21.22			
PR ^c^											0.737	
Negative		377	295			78.25		82	21.75			
Positive		747	591			79.12		156	20.88			
HER-2^ c^											0.047*	
Negative		795	614			77.23		181	22.77			
Positive		327	270			82.57		57	17.43			
Family History of Breast Cancer											0.474	
No		1160	887			76.47		273	23.53			
Yes		11	10			90.91		1	9.09			

^a^, The Non-mas Group Included BCS Cases and Non-surgery Cases; ^b^, The Number of Patients Undergoing This Surgery Divided by All Breast Cancer Cases in Each Row; ^c^, These Data Were Missing for Some Patients Who Did Not Have Surgery, and Those Patients with Missing Data were Excluded from the Data Analysis; *,Statistically Significant; MAS, Mastectomy; BCS, Breast-Conserving Surgery; ER, Estrogen Receptor; PR, Progesterone Receptor; HER-2, Human Epidermal Growth Factor Receptor 2; Cm, Centimetre.

**Table 3:** Independent Predictors of Mastectomy Using Multivariate Logistic Regression Analysis (BCS as the Referenced Group)

Variables	*P-value*	B	OR^a^	95% CI
Age (Ref. 22-39)				
40-59	0.009 *	0.649	1.913	1.176-3.111
60+	0.037 *	0.621	1.862	1.037-3.343
Cancer Stage (Ref. Stage 0)				
Stage 2	0.042 *	0.609	1.839	1.023-3.305
Stage 3	0.021 *	1.001	2.722	1.161-6.381
Tumour Size (Ref. <2 cm)				
2-5 cm	<0.001 *	0.774	2.169	1.419-3.316
>5 cm	0.047 *	0.779	2.180	1.012-4.697
HER-2 (Ref. Negative)				
Positive	0.039 *	0.709	2.031	1.036-3.980

^a^, Adjusted for Age, Marital Status, Fertility History, Cancer Stage, Histology, Tumour Size, Lymph Node Invasion and Expression of HER-2; *, Statistically Significant; B, Regression Coefficient; OR, Odds Ratio; CI, Confidence Interval.

## Discussion

In our study, 76.60% of 1,171 breast cancer patients underwent a mastectomy from 2015 to 2019, demonstrating the wide application of this surgery in China from a single-institution perspective. Despite the fluctuations, the percentage of patients treated with a mastectomy showed a slightly increasing pattern from 70.62% in 2015 to 71.91% in 2019. 

The mastectomy rate in our study was relatively lower than those in previous studies in China (generally greater than 80%) (Zhang et al., 2013; Lu et al., 2015; Huang et al., 2016; Feng et al., 2018). Compared to previous studies, the lower mastectomy rates in the present study could be attributed to the relatively small sample size in ZSXM. It might also reveal the variations in breast cancer treatments at the regional level and institution level in China. A prior study reported that the uptake of mastectomy was lower in hospitals in higher economic areas when compared to those in lower economic areas thanks to the geographical variations in socio-economic status, health care service accessibility, and health education (Zhang et al., 2013). The geographic location of our study, Xiamen, is a high-economic city in eastern China, permitting patients in ZSXM to access more treatment options other than a mastectomy; thus, the mastectomy rate is relatively lower than that in previous multi-centre reports.

Unlike China, BCS is the major surgical procedure used to treat breast cancer in most Western countries, and the percentage of women undergoing mastectomy has been declining (Katipamula et al., 2009; Garcia-Etienne et al., 2012; Albornoz et al., 2015; Heeg et al., 2020). Although the application of unilateral mastectomy was declining, the reconstructive mastectomy and prophylactic mastectomy were increasing, leading to the fluctuation in mastectomy rates (Albornoz et al., 2015). However, in this study, only 2.3% of all women had an immediate reconstruction after mastectomy, which was considerably lower than that in the UK (9%-43%) (Jeevan et al., 2014) and US (10%-27%) (Albornoz et al., 2015). The high rate of mastectomy in this institution was therefore unlikely related to the application of breast reconstruction. This study could not assess the contribution of prophylactic mastectomy to the mastectomy rates due to the lack of this information in the EMRS of this hospital. However, prophylactic mastectomy is generally not recommended by surgeons in China because of controversy over its efficacy (Wong et al., 2017; Huang and Yin, 2018). The impacts of prophylactic mastectomy on mastectomy use in China could be smaller than in Western countries.

In the present study, more than 80% of women with ESBC underwent a mastectomy, and the mastectomy trend for women with ESBC was increasing. This finding might suggest that researchers should be thinking about whether mastectomy is overused for ESBC in China (Zhang et al., 2013; Huang et al., 2016). Since the surgical treatment for ESBC is more “preference-driven”, we cannot come to any precise conclusions without an understanding of patients’ and surgeons’ perspectives about mastectomy use. More research about treatment decision-making among surgeons and patients with ESBC should be performed in China.

Age has shown varied effects on breast cancer surgical management across the previous literature, and most of them have indicated that older age is associated with an increased uptake of mastectomy, which is consistent with our results (Huang et al., 2016; Al-Gaithy et al., 2019; Chen et al., 2019). We found that women who were married and had at least one child were significantly more likely to undergo a mastectomy. Studies suggested that the breast has more cultural significance in relation to lactation rather than appearance in traditional Chinese cultural values (Lam et al., 2005; Killoran and Moyer, 2006; Zhang et al., 2012). Thus, Chinese women might be less concerned about changes in body image caused by a mastectomy. Moreover, due to the unique reproductive pattern in China (implications of the one-child policy from the 1980s to 2015), women might no longer need to breastfeed after giving birth to their one child, which may be another reason for them to accept a mastectomy. 

We found women undergoing mastectomy had a more advanced cancer presentation (a larger tumour, more positive lymph nodes, a more advanced stage, and positive for HER-2). Among these factors, an increased tumour size (2-5 cm), higher stages (stage 2-stage 3) and a positive HER-2 status were independent predictors of a mastectomy. Women with a larger tumour are more likely to have advanced breast cancer, leading to fewer treatment options. In addition, since Chinese women often have a smaller size of the breast, a larger ratio of the tumour-to-breast size impairs their breast appearance, probably promoting their willingness to have the entire breast removed. Women with advanced breast cancer have also been reported to have an increased risk of breast cancer recurrence, and thus women with a larger tumour and higher stage cancer prefer more reassuring managements (i.e., mastectomy) with the expectancy of complete healing. Besides, since being positive for HER-2 is associated with a higher risk of cancer recurrence (Lowery et al., 2012), women with HER-2 positive breast cancer might require more radical and extensive treatments, such as a mastectomy combined with chemotherapy and targeted therapy, to avoid cancer recurrence and to achieve an improved survival rate. 

In this study, however, triple-negative cancer, the presence of ER/PR and a family history of breast cancer did not affect surgical treatments, which contradicted the previously reported correlations(Garcia-Etienne et al., 2012; Zhang et al., 2015; Huang et al., 2016). This discordance might be due to the different distribution of the patients’ characteristics in our study. For example, patients in the present study predominantly had no family history of breast cancer. Further investigation is required to determine whether there is a significant difference in the association of biological subtypes of breast cancer, the presence of ER/PR and a family history of breast cancer with the uptake of mastectomy.

It is estimated that breast cancer incidence in China has accelerated at twice the rate worldwide, especially in rural regions (Fan et al., 2014). The insufficiency of the health care services, the unsatisfactory proportion of women receiving cancer screening and the low rate of the surgeon’s referral to radiotherapy might contribute to the high receipt of mastectomy among Chinese patients (Fan et al., 2014; Song et al., 2018). Sivasubramaniam et al., (2015) compared the surgical treatments for breast cancer between Chinese-American women in the US and Chinese women in China, and found women in China were diagnosed with larger tumours and a more advanced cancer stage. The advanced cancer presentation among women in China was associated with less knowledge about early detection of cancer. China has launched a variety of programs to promote breast cancer screening, however, the proportion of undergoing breast cancer screening is unsatisfactory (Wang et al., 2013; Wu et al., 2020). A study reported the participation rates (about 21.7%) in breast cancer screening in China was lower than those in Western countries (more than 70%), as well as the goal proposed by the World Health Organization (Wang et al., 2013). Wu et al., (2020) surveyed the physicians in China to explore their breast cancer screening beliefs, recommendations and practice, and found that many physicians might not fully aware of current evidence and screening guidelines, which might result in less breast cancer screening. Thus, training programs about screening guideline should be tailored for physicians to enhance their knowledge, which could improve the screening rates among Chinese women. Another reason for increasing mastectomy could be healthcare expenditures. The cost of mastectomy is less than BCS, and thus the receipt of mastectomy might be affected by the patients’ financial considerations (Zhang, 2008). Also, in the US, the incidence of mastectomy has been regarded as an objective measure of the quality of breast cancer care, since it is believed to involve institutional treatment bias, inadequate counselling, and limited access to radiotherapy (Damle et al., 2011). The lack of a similar appraisal provision regarding the quality of breast cancer care could be another reason for the difference in mastectomy use between China and the US.

The findings from the present study were limited to data from a single tertiary institution in eastern China, which might affect their generalizability. More multi-centre studies are needed. We also omitted some important confounding factors that might contribute to the mastectomy, such as the patients’ education level, the patient’s knowledge about breast cancer treatments, and the surgeon’s recommendations of mastectomy. These factors were poorly documented in the medical record system. In addition, we did not analyze the specific types of mastectomy, and additional studies are necessary to evaluate the trends in the uptake of different subtypes of mastectomy, such as modified radical mastectomy, simple mastectomy and nipple-sparing mastectomy. 

This study provides updated data on mastectomy rates, supporting the predominance of this surgery in mainland China. Findings from this study might contribute to understanding the reasons for the wide application of mastectomy in China. Findings suggested the potential overuse of mastectomy among women with ESBC, and highlighted the significance of promoting cancer screening. Since there is a lack of a policy and strategy regarding decision-making about breast cancer treatment in China, these findings could be also used to develop related provisions and interventions to facilitate patients making treatment decisions and screening planning.

## Author Contribution Statement

All authors contributed to the study conception and design. Data collection and analysis were performed by Jing Liu and Dongmei Guo. The first draft of the manuscript was written by Jing Liu. Sharyn Hunter, Jiemin Zhu, Regina Lai Tong Lee and Sally Wai-Chi Chan provided substantial comments on this manuscript. All authors read and approved the final manuscript.

## Data Availability

All data used in this study are available from the corresponding author upon reasonable request.

## References

[B1] Al-Gaithy Z, Yaghmoor B, Koumu M (2019). Trends of mastectomy and breast-conserving surgery and related factors in female breast cancer patients treated at King Abdulaziz University Hospital, Jeddah, Saudi Arabia, 2009-2017: a Retrospective Cohort Study. Ann Med Surg.

[B2] Albornoz C, Matros E, Lee C (2015). Bilateral mastectomy versus breast-conserving surgery for early-stage breast cancer: The Role of Breast Reconstruction. Plast Reconstr Surg.

[B3] Chan P, Choo B, Zhang T (2015). Mastectomy rates remain high in Singapore and are not associated with poorer survival after adjusting for age. SpringerPlus.

[B4] Chen R, You S, Yin Z (2019). Non-doctoral factors influencing the surgical choice of Chinese patients with breast cancer who were eligible for breast-conserving surgery. World J Surg Oncol.

[B5] Chen W, Zheng R, Baade P (2016). Cancer statistics in China, 2015. Ca Cancer J Clin.

[B6] Cutuli B, Dalenc F, Cottu PH (2015). Impact of screening on clinicopathological features and treatment for invasive breast cancer: results of two national surveys. Cancer Radiother.

[B7] Damle S, Teal CB, Lenert JJ (2011). Mastectomy and contralateral prophylactic mastectomy rates: An Institutional Review. Indian J Surg Oncol.

[B8] Fan L, Strasser-Weippl K, Li J-J (2014). Breast cancer in China. Lancet Oncol.

[B9] Feng F, Wei Y, Zheng K (2018). Comparison of epidemiological features, clinicopathological features, and treatments between premenopausal and postmenopausal female breast cancer patients in western China: a retrospective multicentre study of 15,389 female patients. Cancer Med.

[B10] Garcia-Etienne CA, Tomatis M, Heil J (2012). Mastectomy trends for early-stage breast cancer: a report from the EUSOMA multi-institutional European database. Eur J Cancer.

[B11] Habermann EB, Abbott A, Parsons HM (2010). Are mastectomy rates really increasing in the United States?. J Clin Oncol.

[B12] Harding C, Pompei F, Burmistrov D (2019). Use of mastectomy for overdiagnosed breast cancer in the United States: Analysis of the SEER 9 Cancer Registries. J Cancer Epidemiol.

[B13] Heeg E, Jensen MB, Mureau MAM (2020). Breast-contour preserving procedures for early-stage breast cancer: a population-based study of the trends, variation in practice and predictive characteristics in Denmark and the Netherlands. Breast Cancer Res Treat.

[B14] Huang N, Liu M, Chen J (2016). Surgical management of breast cancer in China: A 15-year single-centre retrospective study of 18,502 patients. Medicine.

[B15] Huang X, Yin Y (2018). Updates of Chinese Society of Clinical Oncology (CSCO) guideline for breast cancer in 2018 [The article was originally published in Chinese language]. Chin Med J.

[B16] Jeevan R, Cromwell DA, Browne JP (2014). Findings of a national comparative audit of mastectomy and breast reconstruction surgery in England. J Plast Reconstr Aesthet Surg.

[B17] Katipamula R, Degnim A, Hoskin T (2009). Trends in mastectomy rates at the Mayo Clinic Rochester: Effect of Surgical Year and Preoperative Magnetic Resonance Imaging. J Clin Oncol.

[B18] Killoran M, Moyer A (2006). Surgical treatment preferences in Chinese-American women with early-stage breast cancer. Psychooncology.

[B19] Lam W, Fielding R, Ho E (2005). Surgeon’s recommendation, perceived operative efficacy and age dictate treatment choice by Chinese women facing breast cancer surgery. Psychooncology.

[B20] Linos E, Spanos D, Rosner B (2008). Effects of reproductive and demographic changes on breast cancer incidence in China: a modeling analysis. J Natl Cancer Inst.

[B21] Lowery AJ, Kell MR, Glynn RW (2012). Locoregional recurrence after breast cancer surgery: a systematic review by receptor phenotype. Breast Cancer Res Treat.

[B22] Lu JJ, Li HA, Xiong Y (2015). Breast cancer inpatients undergoing mastectomy from a Hospital in Guangzhou, China: A Retrospective Analysis 2004-2013. Asian Pac J Cancer Prev.

[B23] Sivasubramaniam P, Zhang B, Zhang Q (2015). Breast cancer disparities: A Multicentre Comparison of Tumour Diagnosis, Characteristics, and Surgical Treatment in China and the U. S. Oncologist.

[B24] Song S, Yuan B, Zhang L (2018). Increased inequalities in health resource and access to health care in rural China. Int J Environ Res Public Health.

[B25] Wang B, He M, Wang L (2013). Breast cancer screening among adult women in China, 2010. Prev Chronic Dis.

[B26] Wong SM, Freedman RA, Sagara Y (2017). Growing use of contralateral prophylactic mastectomy despite no improvement in long-term survival for invasive breast cancer. Ann Surg.

[B27] Wong WJ, Mosiun JA, Hidayati Z (2019). Low Breast Conserving Surgery (BCS) rates in public hospitals in Malaysia: The effect of stage and ethnicity. Breast J.

[B28] Wu T-Y, Raghunathan V, Shi J (2020). Improving the outcomes of breast cancer in China: Physicians’ Beliefs, Recommendations, and Practices for Breast Cancer Screening. Asian Pac J Cancer Care.

[B29] Yip CH, Cazap E, Anderson BO (2011). Breast cancer management in middle-resource countries (MRCs): consensus statement from the Breast Health Global Initiative. Breast J.

[B30] Zhang B (2008). Current situation and development of breast conserving surgery for breast cancer [The article was originally published in Chinese language]. Chin J Pract Surg.

[B31] Zhang B, Sivasubramaniam P, Zhang Q (2015). Trends in radical surgical treatment methods for breast malignancies in China: A Multicentre 10-Year Retrospective Study. Oncologist.

[B32] Zhang B, Song Q, Zhang B (2013). A 10-year (1999-2008) retrospective multi-centre study of breast cancer surgical management in various geographic areas of China. Breast J.

[B33] Zhang L, Jiang M, Zhou Y (2012). Survey on breast cancer patients in China toward breast-conserving surgery. Psychooncology.

